# Supplementary material for: Bis(maltolato)oxovanadium(IV) Induces Angiogenesis via Phosphorylation of VEGFR2
Source: Int J Mol Sci. 2020 Jun 30;21(13):4643. doi: 10.3390/ijms21134643 (PMC7370103; doi:10.3390/ijms21134643)
Supplement: Supplementary file 1 [file ijms-21-04643-s001.pdf]

Supplemental material

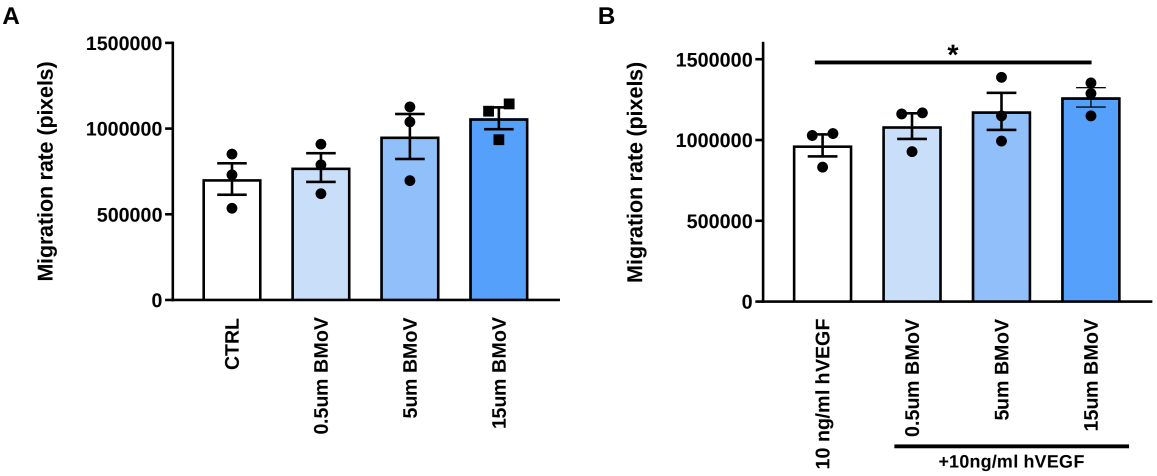

Figure S1. Quantification of HUVECs scratch-wound healing after treatment with either (**A**) BMOV alone in different concentrations (0.5, 5, 15 µM), or (**B**) different concentrations of BMOV (0.5, 5, 15 µM) supplemented with 10 ng/mL VEGF-A. Datapoints represent averages obtained from 3 independent experiments and are presented as mean ± SEM. **p* < 0.05 by two-sided Student t test.

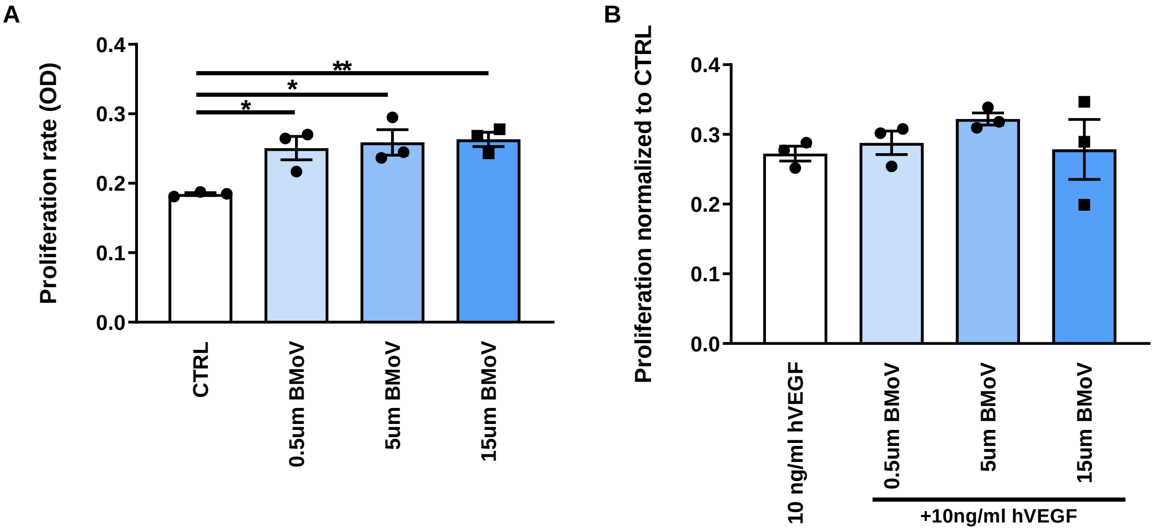

Figure S2. Quantification of HUVECs proliferation after treatment with either (**A**) BMOV alone in different concentrations (0.5, 5, 15 µM), or (**B**) different concentrations of BMOV (0.5, 5, 15 µM) supplemented with 10 ng/mL VEGF-A. Datapoints represent averages obtained from 3 independent experiments and are presented as mean ± SEM. **p* < 0.05; ***p* < 0.01 by two-sided Student t test.

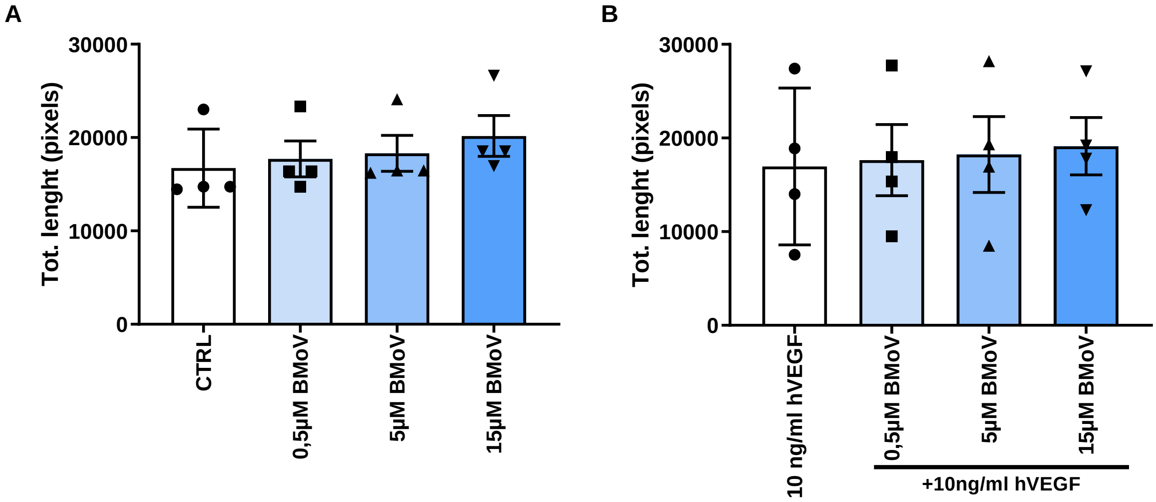

Figure S3. Quantification of HUVECs tube formation after treatment with either (**A**) BMOV alone in different concentrations (0.5, 5, 15 µM), or (**B**) different concentrations of BMOV (0.5, 5, 15 µM) supplemented with 10 ng/mL VEGF-A. Datapoints represent averages obtained from 3 independent experiments and are presented as mean ± SEM. *$p < 0.05$ by two-sided Student t test.

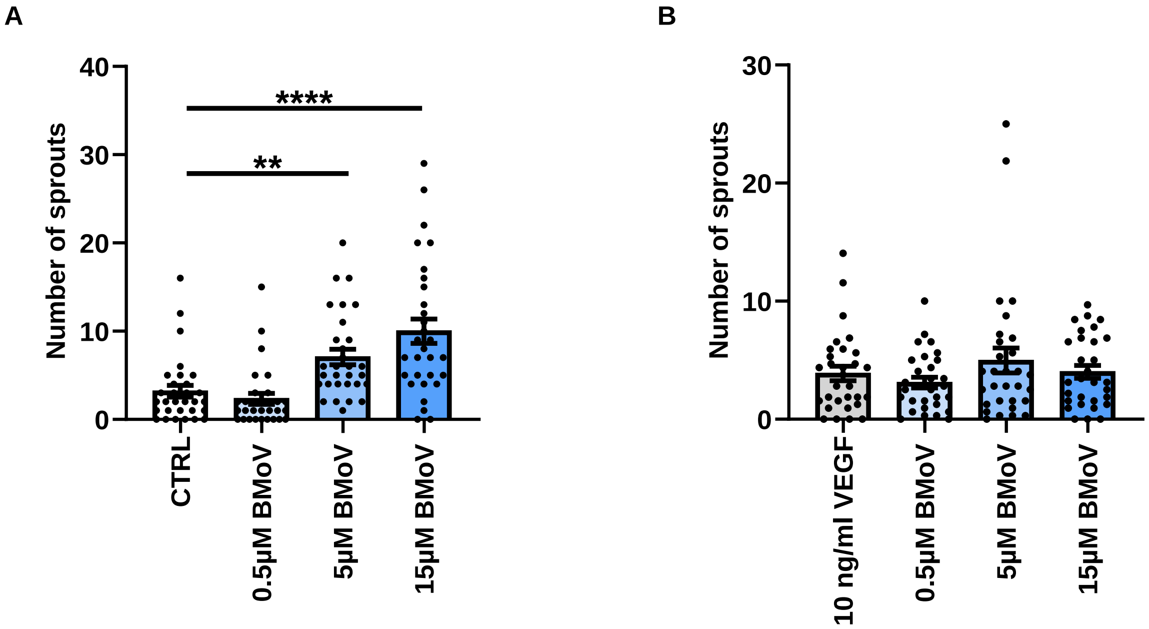

Figure S4. Quantification of neovessel sprouts after treatment with either (**A**) BMOV alone in different concentrations (0.5, 5, 15 µM), or (**B**) different concentrations of BMOV (0.5, 5, 15 µM) supplemented with 10 ng/mL VEGF-A in an ex vivo aortic ring assay. Datapoints are presented as mean±SEM. ****$p < 0.0001$ by two-sided Student t test.

© 2020 by the authors. Submitted for possible open access publication under the terms and conditions of the Creative Commons Attribution (CC BY) license (http://creativecommons.org/licenses/by/4.0/).
